# High-content, arrayed compound screens with rhinovirus, influenza A virus and herpes simplex virus infections

**DOI:** 10.1038/s41597-022-01733-4

**Published:** 2022-10-08

**Authors:** Dominik Olszewski, Fanny Georgi, Luca Murer, Vardan Andriasyan, Fabien Kuttler, Anthony Petkidis, Robert Witte, Artur Yakimovich, Lucy Fischer, Alina Rozanova, Yohei Yamauchi, Gerardo Turcatti, Urs F. Greber

**Affiliations:** 1 Department of Molecular Life Sciences, University of Zurich (UZH), Winterthurerstrasse 190, 8057 Zurich, Switzerland; 2 Biomolecular Screening Facility, School of Life Sciences, Ecole Polytechnique Fédérale (EPFL) de Lausanne, Station 15, Lausanne, 1015 Switzerland; 3 Center for Advanced Systems Understanding (CASUS), Helmholtz Center Dresden-Rossendorf, Untermarkt 20, 82026 Görlitz, Germany; 4 Bladder Infection and Immunity Group (BIIG), Department of Renal Medicine, Division of Medicine, University College London, Royal Free Hospital Campus, London, NW3 2PF United Kingdom; 5 University of Bristol, Bristol, BS8 1TH United Kingdom; 6 Present Address: Institute of Pharmaceutical Sciences, ETH Zurich, Vladimir-Prelog-Weg 1-5/10, 8093 Zurich, Switzerland

**Keywords:** High-throughput screening, Viral infection

## Abstract

Viruses are genetically and structurally diverse, and outnumber cells by orders of magnitude. They can cause acute and chronic infections, suppress, or exacerbate immunity, or dysregulate survival and growth of cells. To identify chemical agents with pro- or antiviral effects we conducted arrayed high-content image-based multi-cycle infection screens of 1,280 mainly FDA-approved compounds with three human viruses, rhinovirus (RV), influenza A virus (IAV), and herpes simplex virus (HSV) differing in genome organization, composition, presence of an envelope, and tropism. Based on Z’-factors assessing screening quality and Z-scores ranking individual compounds, we identified potent inhibitors and enhancers of infection: the RNA mutagen 5-Azacytidine against RV-A16; the broad-spectrum antimycotic drug Clotrimazole inhibiting IAV-WSN; the chemotherapeutic agent Raltitrexed blocking HSV-1; and Clobetasol enhancing HSV-1. Remarkably, the topical antiseptic compound Aminacrine, which is clinically used against bacterial and fungal agents, inhibited all three viruses. Our data underscore the versatility and potency of image-based, full cycle virus propagation assays in cell-based screenings for antiviral agents.

## Abstract (summary)

**Table:** 

Measurement(s)	transmission of virus • drug toxicity
Technology Type(s)	fluorescence microscopy
Factor Type(s)	virus genotype • cell line • incubation temperature • number of cells seeded/ seeding density • inoculation time • virus input FFU • drug concentration
Sample Characteristic - Organism	Homo sapiens • Rhinovirus A • Influenza A virus • Human alphaherpesvirus 1
Sample Characteristic - Environment	epithelium
Sample Characteristic - Location	Switzerland

## Background & Summary

Viruses pose major threats to human health beyond SARS-CoV-2, the causative agent of COVID-19. The World Health Organization lists at least 15 potentially epidemic or pandemic infectious diseases, mostly viral^1^. While vaccination has been very effective to limit many viral diseases, important viruses lack approved vaccines. Considering that viruses require host and viral factors for replication and maintenance, it is likely that all viruses are susceptible to antiviral compounds. A majority of approved antiviral agents targets viral factors, and is subject to evasion by mutations^2^. Hence, the search for host targeting antivirals is increasing^3,4^. One approach is to revisit compounds approved for human use in nonviral disease, and assess their anti-viral potency for repurposing^5–9^.

We present pro- and antiviral effects of mostly FDA-approved chemicals on three different human viruses: rhinovirus (RV) type A16, influenza A virus (IAV) H1N1 WSN strain, and herpes simplex virus (HSV) type 1 (Table 1). RVs belong to the family *Picornaviridae* and comprise more than 160 genotypes^10^. Their particles are non-enveloped and have a positive-sense single-stranded (+)ssRNA genome of about 7.2 kb^11^. RVs are highly prevalent and infect the upper respiratory tract, typically with common cold symptoms and exacerbated disease in patients suffering from chronic respiratory conditions^12–14^. Currently, there are no approved vaccines or antiviral therapies against RVs^15^. Influenza viruses belong to the *Orthomyxoviridae* family with four genera A, B, C, and D, and cause seasonal and sporadic flu pandemics^16^. They contain a 10–14.6 kb segmented negative-sense (−)ssRNA genome in enveloped particles. Influenza viruses are clinically treated by direct-acting antivirals against the cap-dependent endonuclease or the neuraminidase, or antibodies against the hemagglutinin, albeit subject to viral evasion^17^. HSV-1 is an alphaherpesvirus causing recurrent cutaneous lesions. It forms enveloped particles containing an icosahedral protein capsid with a double-stranded DNA genome of about 150 kb delivered and replicated in the cell nucleus^18,19^. Acyclovir and some of its derivatives are used against HSV but not against the cancer-causing gamma-herpesvirus Kaposi’s Sarcoma-associated herpesvirus (KSHV), although Ganciclovir has been used against Epstein-Barr virus (EBV)^20–23^.

**Table 1:** Characteristics and screening conditions for the different viruses tested against the PCL compounds. The viruses differ in their genome composition, size, envelopment, tropism, and plaque shape. The screening conditions were optimized for each virus individually. FFU = focus forming unit, GFP = green fluorescent protein, NP = nucleoprotein, BafA1 = Bafilomycin A1, GCV = Ganciclovir.

		RV-A16	IAV-H1N1 (WSN)	HSV-1
Characteristics	Genome	Linear (+)ssRNA	Segmented (−)ssRNA	Linear dsDNA
	Genome/Virion	7.2 kb/30 nm	13.5 kb/100 nm	152 kbp/130 nm
	Envelope	no	yes	yes
	Tropism	Upper respiratory tract	Respiratory tract	Epithelial mucosa, neuronal cells
Screening conditions	Read-out	GFP	α-NP antibody staining	GFP
	Plaque shape	Round, comet	Comet	Round, comet
	Positive control	0.6 nM BafA1	1.5 nM BafA1	25 µM Ganciclovir
	Negative control	0.0125% DMSO	0.0125% DMSO	0.025% DMSO
	Number of cells per well seeded	6,000 HOG	10,000 A549	8,000 A549
	Inoculation FFU per well	30	25	50
	Infection time	48 h	24 h	26 h
	Infection temperature	33.5 °C	37 °C	37 °C

**Fig. 1:**
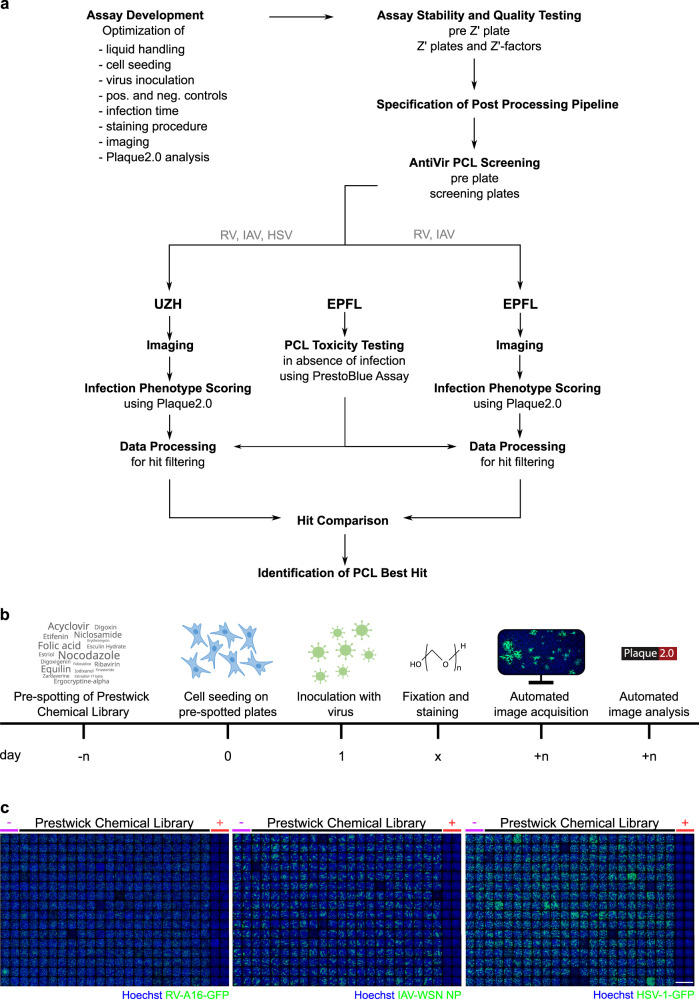
Screening pipeline. (**a**) Prior to screen execution, several assay parameters were optimized. Stability and quality were verified with pre-plates and Z’-plates. The subsequent screening was followed by image acquisition and analysis at two independent institutions UZH and EPFL. Identified hits were compared and best hits were selected. (**b**) Timeline for the wet-lab pipeline. A549 or HOG cells were seeded on 384-well plates pre-spotted with PCL compounds and control compounds through an Echo acoustic liquid handling system, yielding a final concentration of 1.25 μM upon cell seeding in 80 μl. On the next day, the adherent cells were inoculated with virus, PFA-fixed after 48 h for RV, 34 h for IAV, and 26 h for HSV-1, and stained with Hoechst 33342 and antibody, if necessary. Plates were then imaged using a high-throughput epifluorescence microscope, and analyzed with the Plaque2.0 software. (**c**) Exemplary overviews of one screening plate for each virus screen. Individual images of each well were stitched yielding the overviews. First two columns of each plate contained DMSO control solvent, the following 20 columns PCL compounds, and the last two columns the positive controls (BafA1 for RV and IAV, and GCV for HSV-1). Scale bar represents 10 mm.

**Fig. 2:**
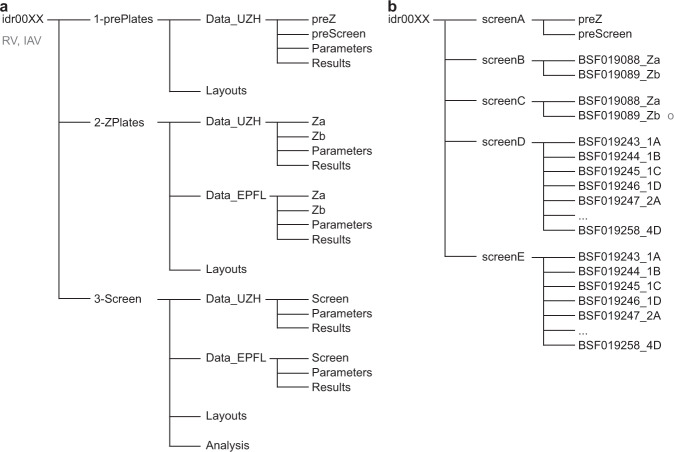
Structure of data available at the Image Data Resource. (**a**,**c**) Data provided for download are organized in three folders 1-prePlates, 2-ZPlates, 3-Screen, and contain parameters, result files, images, and analyses of the plates. (**b**,**d**) The data for viewing are divided into five screens, where screenA contains the pre-plates, screenB and -C contain the Z’-plates imaged at UZH and EPFL, respectively, and screenD and -E hold the images acquired of the screening plates at UZH and EPFL, respectively. For HSV (**d**), images of the screening plates can be accessed under screenC. Note that image acquisition and analyses for HSV-1 were performed at UZH only, not at EPFL.

Here, we conducted three parallel image-based virus infection screens on a platform that we previously used for adenovirus^5^. We employed the Prestwick Chemical Library (PCL) containing 1,280 mostly FDA-approved compounds targeting a broad range of diseases, including cancer and infections^5,24,25^. Cells were seeded onto the pre-spotted compounds at 1.25 μM final compound concentration. A single concentration of 1.25 μM risks to miss those compounds that have an antiviral efficacy <1.25 µM and toxicity >1.25 µM. We estimated, however, that the number of such compounds would be very low, based on our earlier image-based screen with coronavirus 229E-GFP using a library of 5440 mostly FDA approved compounds (including the PCL compounds) at 1.67 µM^9^. This screen yielded only five of 5440 compounds (0.1%) with tox50 > 1.25 µM and EC50 < 0.25 µM. Assuming a similar frequency of low EC50 and high tox50 compounds in the PCL, this would amount to 1–2 compounds. Seeded cells were subsequently infected for several rounds of replication, fixed and analyzed by Plaque2.0 software^26^, followed by scoring the number of infected and total cell nuclei, infection index (ratio of infected nuclei over total nuclei), number of plaques (fluorescent foci), and integrated signal intensity of infection markers (Table 1, Fig. 1, and Supplementary Fig. 1). Four replicate screens with RV and IAV demonstrated high quality indicated by mean Z’-factors of 0.13–0.53 determined at two institutions, UZH and EPFL, while the HSV-1 screen was performed in three replicates and imaged and analyzed at one institution. All the raw and scored data are available at the Image Data Resource (IDR, idr.openmicroscopy.org) with the accession numbers idr0128 (IAV)^27^, idr0129 (RV)^28^, and idr0130 (HSV)^29^. The structure of the data is described in Fig. 2 and scores in Tables 2 and 3.

**Table 2:** Representation of the Z’-factor plates. To assess the quality of the screening platform, Z’-factors were determined in dedicated plates before conducting the drug screens. For each virus, two plates with 192 technical replicates of each positive and negative control were infected, and Z’-factors determined for the five screen read-outs by independent imaging at UZH and EPFL (RV and HSV-1 only at UZH) using the formula given in Eq. (1) for 3σ and 2σ.

RV	Plate	UZH					UZH				
Barcode		3 sigma					2 sigma				
		numberOfNuclei	numberOfInfectedNuclei	infectionIndex	numberOfPlaques	totalVirusIntensity	numberOfNuclei	numberOfInfectedNuclei	infectionIndex	numberOfPlaques	totalVirusIntensity
BSF019007	Za	−12.59	0.50	0.51	0.53	0.49	−8.06	0.67	0.67	0.68	0.66
Mean		−12.59	0.50	0.51	0.53	0.49	−8.06	0.67	0.67	0.68	0.66
**IAV**		**UZH**					**UZH**				
**Barcode**	**Plate**	**3 sigma**					**2 sigma**				
		**numberOfNuclei**	**numberOfInfectedNuclei**	**infectionIndex**	**numberOfPlaques**	**totalVirusIntensity**	**numberOfNuclei**	**numberOfInfectedNuclei**	**infectionIndex**	**numberOfPlaques**	**totalVirusIntensity**
BSF019088	Za	−0.15	0.14	0.14	0.32	0.30	0.23	0.42	0.43	0.55	0.53
BSF019089	Zb	−0.22	0.18	0.13	0.42	0.38	0.18	0.46	0.42	0.61	0.58
Mean		−0.19	0.16	0.13	0.37	0.34	0.21	0.44	0.42	0.58	0.56
		**EPFL**					**EPFL**				
**Barcode**	**Plate**	**3 sigma**					**2 sigma**				
		**numberOfNuclei**	**numberOfInfectedNuclei**	**infectionIndex**	**numberOfPlaques**	**totalVirusIntensity**	**numberOfNuclei**	**numberOfInfectedNuclei**	**infectionIndex**	**numberOfPlaques**	**totalVirusIntensity**
BSF019088	Za	−0.02	0.39	0.36	0.42	0.41	0.32	0.59	0.57	0.61	0.61
BSF019089	Zb	−0.08	0.36	0.30	0.50	0.50	0.28	0.58	0.53	0.67	0.66
Mean		−0.05	0.37	0.33	0.46	0.45	0.30	0.58	0.55	0.64	0.64
**HSV**		**UZH**					**UZH**				
**Barcode**	**Plate**	**3 sigma**					**2 sigma**				
		**numberOfNuclei**	**numberOfInfectedNuclei**	**infectionIndex**	**numberOfPlaques**	**totalVirusIntensity**	**numberOfNuclei**	**numberOfInfectedNuclei**	**infectionIndex**	**numberOfPlaques**	**totalVirusIntensity**
BSF020768	Za	−8.32	0.04	0.20	0.37	−0.01	−5.21	0.36	0.47	0.58	0.32
BSF020769	Zb	−23.01	0.25	0.21	0.38	0.21	−15.01	0.50	0.47	0.59	0.47
Mean		−15.67	0.15	0.20	0.37	0.10	−10.11	0.43	0.47	0.58	0.40

**Table 3:** Compilation of Z’-factors derived from screening plates. Z’-factors are used to assess the quality of the screens. Based on 32 technical replicates of each positive and negative control compounds the Z’-factors were determined for each virus and screening plate considering the five imaging read-outs determined independently at UZH and EPFL using the formula given in Eq. (1) for 3σ. Note HSV-1 was assessed at UZH only.

RV	Plate	UZH					EPFL				
Barcode		3 sigma					3 sigma				
		numberOfNuclei	numberOfInfectedNuclei	infectionIndex	numberOfPlaques	totalVirusIntensity	numberOfNuclei	numberOfInfectedNuclei	infectionIndex	numberOfPlaques	totalVirusIntensity
BSF019072	1A	−5.63	0.42	0.32	0.59	0.37	−2.32	0.48	0.17	0.61	0.48
BSF019073	1B	−4.04	0.40	0.57	0.56	0.43	−2.45	0.44	0.19	0.54	0.49
BSF019074	1C	−8.64	0.55	0.50	0.62	0.51	−4.95	0.58	0.41	0.64	0.63
BSF019075	1D	−5.11	0.36	0.51	0.60	0.33	−2.13	0.38	0.19	0.58	0.42
BSF019076	2A	−9.35	0.37	0.52	0.69	0.55	−14.68	0.47	0.35	0.69	0.55
BSF019077	2B	−6.94	0.41	0.64	0.66	0.54	−5.16	0.49	0.36	0.66	0.49
BSF019078	2C	−3.83	0.15	0.58	0.62	0.54	−4.15	0.40	0.30	0.65	0.49
BSF019079	2D	−5.83	0.43	0.56	0.65	0.52	−5.28	0.51	0.37	0.70	0.54
BSF019080	3A	−71.51	0.39	0.58	0.70	0.51	−18.34	0.48	0.22	0.62	0.44
BSF019081	3B	−66.04	0.40	0.45	0.59	0.55	−15.04	0.50	0.31	0.64	0.56
BSF019082	3C	−15.63	−0.03	0.62	0.63	0.58	−21.98	0.26	0.23	0.63	0.44
BSF019083	3D	−12.46	0.54	0.37	0.55	0.53	−14.19	0.61	0.34	0.66	0.55
BSF019084	4A	−0.53	0.70	0.66	0.71	0.74	−0.37	0.71	0.62	0.71	0.58
BSF019085	4B	−0.29	0.69	0.54	0.71	0.69	−0.32	0.70	0.56	0.77	0.57
BSF019086	4C	−0.31	0.72	0.44	0.71	0.71	−0.46	0.72	0.60	0.74	0.52
BSF019087	4D	−0.09	0.68	0.60	0.66	0.60	−0.07	0.64	0.54	0.66	0.49
Mean		−13.51	0.45	0.53	0.64	0.54	−6.99	0.52	0.36	0.66	0.52
**IAV**		**UZH**					**EPFL**				
**Barcode**	**Plate**	**3 sigma**					**3 sigma**				
		**numberOfNuclei**	**numberOfInfectedNuclei**	**infectionIndex**	**numberOfPlaques**	**totalVirusIntensity**	**numberOfNuclei**	**numberOfInfectedNuclei**	**infectionIndex**	**numberOfPlaques**	**totalVirusIntensity**
BSF019243	1A	−0.77	0.10	0.31	−0.03	−0.03	−1.99	0.09	−0.01	0.16	0.18
BSF019244	1B	−1.25	0.29	0.36	0.13	0.08	−4.53	0.23	0.14	0.17	0.09
BSF019245	1C	−0.71	0.39	0.42	0.24	0.29	−3.48	0.32	0.22	0.21	0.19
BSF019246	1D	−1.19	0.28	0.33	0.09	−0.04	−3.55	0.21	−0.08	0.25	0.16
BSF019247	2A	−2.39	0.34	0.40	0.13	0.16	−4.77	0.28	0.45	0.24	0.29
BSF019248	2B	−0.89	0.28	0.47	0.21	0.20	−11.23	0.23	0.38	0.29	0.28
BSF019249	2C	−0.95	0.42	0.37	0.22	0.25	−3.26	0.36	0.33	0.27	0.34
BSF019250	2D	−1.41	0.25	0.43	0.25	0.23	−1.64	0.18	0.22	0.22	0.18
BSF019251	3A	−2.19	0.36	0.37	0.32	0.33	−7.84	0.19	0.32	0.30	0.31
BSF019252	3B	−1.82	0.02	0.03	−0.21	−0.22	−2.48	0.00	0.19	−0.12	−0.12
BSF019253	3C	−1.79	0.10	0.40	−0.05	−0.17	−0.87	0.07	0.36	−0.03	−0.12
BSF019254	3D	−0.71	0.37	0.31	0.16	0.17	−2.59	0.24	0.20	0.10	0.17
BSF019255	4A	−0.64	0.14	0.32	−0.05	−0.03	−0.46	0.13	0.39	0.09	0.12
BSF019256	4B	−1.02	0.26	0.48	0.27	0.24	−0.05	0.26	0.44	0.21	0.19
BSF019257	4C	−1.45	0.30	0.56	0.23	0.13	−0.58	0.32	0.37	0.27	0.22
BSF019258	4D	−0.79	0.17	0.28	0.02	0.05	−0.59	0.07	0.43	0.10	0.09
Mean		−1.25	0.25	0.37	0.12	0.10	−3.12	0.20	0.27	0.17	0.16
**HSV**		**UZH**									
		**3 sigma**									
**Barcode**	**Plate**	**numberOfNuclei**	**numberOfInfectedNuclei**	**infectionIndex**	**numberOfPlaques**	**totalVirusIntensity**					
BSF020893	1A	−7.87	−0.09	−0.17	−0.18	−0.14					
BSF020897	1B	−8.02	−0.08	−0.12	−0.03	−0.13					
BSF020901	1C	−7.10	0.38	0.38	0.44	0.38					
BSF020905	1D	−29.20	0.25	0.34	0.41	0.27					
BSF020894	2A	−9.27	0.69	0.64	0.67	0.57					
BSF020898	2B	−1.21	0.71	0.77	0.74	0.67					
BSF020902	2C	−2.05	0.71	0.71	0.70	0.68					
BSF020906	2D	−3.20	0.65	0.71	0.69	0.57					
BSF020895	3A	−2.77	0.46	0.44	0.64	0.49					
BSF020899	3B	−2.35	0.39	0.35	0.57	0.33					
BSF020903	3C	−16.94	0.41	0.46	0.65	0.44					
BSF020907	3D	−9.03	0.27	0.24	0.56	0.24					
Mean		−8.25	0.40	0.40	0.49	0.37					

**Fig. 3:**
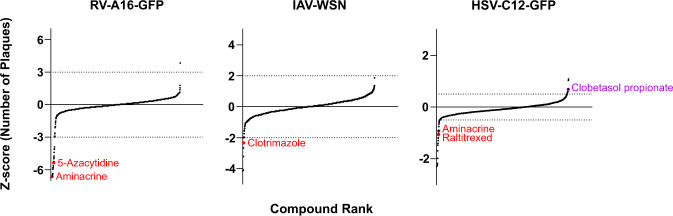
Compound ranking according to Z-scores for each virus tested. Ranked compound scores for the parameter ‘number of plaques’ are displayed. Top hits are highlighted (red = inhibitors; purple = enhancers). Dotted lines indicate the Z-score cut-off for analysis RV (−3), for IAV-WSN (−2) and for HSV-1 (−0.5). A Z-score of −3 or less for a particular compound means that this value was less than the mean of the negative controls minus three standard deviations. Note that Aminacrine inhibited IAV-WSN infection but did not score below the Z-score cut-off of −2 in the screen.

Upon exclusion of toxic compounds, the highest scoring antiviral agents were 5-Azacytidine for RV, Clotrimazole for IAV, and Raltitrexed for HSV, while Clobetasol-propionate enhanced HSV-1 infection transmission, as indicated by Z-score analyses and dose-response validation tests (Fig. 3, Table 4, Supplementary Fig. 1). Aminacrine (9-aminoacridine) scored as top hit for RV, HSV, and was previously reported for AdV-C2^5^. We found that Aminacrine, Raltitrexed, 5-Azacytidine, Clobestasol, and Clotrimazole applied in the micromolar range had no significant effect on the induction of phospholipidosis, unlike the positive control compounds Amiodarone and Ammonium chloride (Supplementary Fig. 2), in agreement with the notion that the latter compounds lead to accumulation of phospholipids in lysosomes^30,31^. In summary, while vaccination is the gold standard in viral disease prevention, not all people can be vaccinated, and disease progression may be rapid and unaccessible to vaccination, which requires alternative treatment options. Direct-acting antivirals raise rapid viral resistance, unless applied in combination treatment, but agents targeting host functions essential for virus propagation offer a vast range of opportunities for novel treatment, also in the context of drug repurposing.

**Table 4:** Top hits with effects on RV, IAV, or HSV infection. The summary includes positive and negative controls. A compound was labelled toxic if scored as such by the PrestoBlue toxicity assay or by the Z-score for the parameter ‘nuclei’ in the screens. Mean read-outs were calculated by averaging four biological replicates (three for HSV) for PCL compounds, and 32 technical replicates over four biological replicates (three for HSV) for positive and negative controls. Normalized values are mean read-outs relative to the mean of the corresponding positive control over all replicates. Compounds that showed low toxicity and high inhibitory or enhancing effects on viral infection were selected as best hits depending on their clinical potential.

Virus	PCL_ID	compoundIdentifier	Compound	Group	Toxic		Mean read-outs					Normalized mean read-outs					Hit					
						Analysis	Nuclei	Infected nuclei	Infection index	Plaques	Virus intensity [AU]	Nuclei	Infected nuclei	Infection index	Plaques	Virus intensity [AU]	Nuclei	Infected nuclei	Infection index	Plaques	Virus intensity [AU]	Effect
RV	DMSO	DMSO	DMSO	neg. ctr.	no	UZH	10,822	2,828	0.26	30.04	4.21E + 09	1.00	1.00	1.00	1.00	1.00	—	—	—	—	—	—
						EPFL	5,547	1,440	0.29	62.64	1.42E + 09	1.00	1.00	1.00	1.00	1.00						
	BafA	BafA	BafA	pos. ctr.	no	UZH	9,096	69	0.01	0.50	3.04E + 09	0.84	0.02	0.03	0.02	0.72	—	—	—	—	—	Inhibitor
						EPFL	4,471	10	0.00	0.38	9.71E + 08	0.41	0.00	0.01	0.01	0.23						
	Prestw-1717	452	Aminacrine	PCL	yes	UZH	8,145	415	0.05	0.25	3.11E + 09	0.75	0.15	0.19	0.01	0.74	no	yes	yes	yes	yes	Inhibitor
						EPFL	4,768	188	0.04	1.50	1.01E + 09	0.44	0.07	0.16	0.05	0.24	no	yes	yes	yes	yes	
	Prestw-388	1157	5-Azacytidine	PCL	yes	UZH	9,195	672	0.07	6.00	3.28E + 09	0.85	0.24	0.27	0.20	0.78	no	yes	yes	yes	yes	Inhibitor
						EPFL	6,015	295	0.05	3.25	1.06E + 09	0.56	0.10	0.19	0.11	0.25	no	no	no	no	no	
	Prestw-787	1078	Merbromin	PCL	no	UZH	12,267	7,148	0.58	47.25	5.81E + 09	1.13	2.53	2.22	1.57	1.38	no	yes	yes	yes	yes	Enhancer
						EPFL	6,035	1,511	0.29	64.75	1.45E + 09	0.56	0.53	1.09	2.16	0.34	no	no	no	no	no	
IAV	DMSO	DMSO	DMSO	neg. ctr.	no	UZH	9,044	1,746	0.19	24.66	4.45E + 09	1.00	1.00	1.00	1.00	1.00	—	—	—	—	—	—
						EPFL	7,977	1,348	0.17	33.66	1.74E + 09	1.00	1.00	1.00	1.00	1.00						
	BafA	BafA	BafA	pos. ctr.	no	UZH	7,782	17	0.00	0.34	2.95E + 09	0.86	0.01	0.01	0.01	0.66	—	—	—	—	—	Inhibitor
						EPFL	7,076	11	0.00	0.38	1.25E + 09	0.89	0.01	0.01	0.01	0.72						
	Prestw-267	631	Clotrimazole	PCL	no	UZH	9,173	481	0.05	10.75	3.36E + 09	1.01	0.28	0.28	0.44	0.75	no	yes	yes	yes	yes	Inhibitor
						EPFL	7,432	410	0.06	12.25	1.39E + 09	0.93	0.30	0.33	0.36	0.80	no	no	no	no	no	
	Prestw-491	850	Metixene hydrochloride	PCL	no	UZH	8,213	2,008	0.24	26.00	5.25E + 09	0.91	1.15	1.26	1.05	1.18	no	no	no	no	yes	Enhancer
						EPFL	7,838	1,571	0.20	38.25	1.80E + 09	0.98	1.17	1.17	1.14	1.03	no	no	no	no	no	
HSV	DMSO	DMSO	DMSO	neg. ctr.	no	UZH	22,286	10,532	0.49	66.06	4.11E + 09	1.00	1.00	1.00	1.00	1.00	—	—	—	—	—	—
	GCV	GCV	GCV	pos. ctr.	no	UZH	20,270	475	0.02	0.77	1.50E + 09	0.91	0.05	0.05	0.01	0.37	—	—	—	—	—	Inhibitor
	Prestw-1717	452	Aminacrine	PCL	no	UZH	18,402	4,467	0.24	39.33	3.06E + 09	0.83	0.42	0.50	0.60	0.75	yes	yes	yes	yes	yes	Inhibitor
	Prestw-1793	525	Raltitrexed	PCL	no	UZH	16,108	3,562	0.22	35.33	2.67E + 09	0.72	0.34	0.45	0.53	0.65	yes	yes	yes	yes	yes	Inhibitor
	Prestw-655	963	Halcinonide	PCL	no	UZH	15,912	13,749	0.85	83.67	5.45E + 09	0.71	1.31	1.75	1.27	1.33	yes	no	yes	no	yes	Enhancer
	Prestw-781	1072	Clobetasol propionate	PCL	yes	UZH	16,267	14,587	0.89	85.67	5.31E + 09	0.73	1.39	1.82	1.30	1.29	yes	yes	yes	yes	yes	Enhancer

## Methods

### Cell culture

A549 cells (human adenocarcinoma alveolar basal epithelial cells) were purchased from the American Type Culture Collection (ATCC, Manassas, USA), and HeLa Ohio cells (HOG, human cervix carcinoma epithelial cells) were obtained from L. Kaiser (Central Laboratory of Virology, University Hospital Geneva, Switzerland) and used as described^32,33^. Cells were cultured at 95% humidity, 37 °C, 5% CO_2_ in high glucose Dulbecco Modified Eagle Medium (DMEM; Thermo Fisher Scientific, Waltham, USA) supplemented with 7.5% fetal bovine serum (FBS; Invitrogen, Carlsbad, USA), 1% L-glutamine (Sigma-Aldrich, St. Louis, USA), and 1% penicillin streptomycin (Sigma-Aldrich, St. Louis, USA). Cells were split regularly upon reaching 90% of confluence by washing with PBS followed by Trypsin-EDTA (Sigma-Aldrich, St. Louis, USA) treatment, not surpassing a passage number of 20.

### Viruses

RV-A16-GFP was kindly provided by Dr. James Gern (University of Wisconsin–Madison, Madison, USA). The virus was expanded by inoculation of HeLa Ohio cells with a lysate from cells infected at 33.5 °C overnight. At occurrence of cytopathic effect (CPE) in 80–90% of cells, the cells were harvested and pelleted. Lysis was performed by three freeze and thaw cycles, and subsequent addition of 1% v/v NP40 and use of a Dounce homogenizer. The lysate was centrifuged at 2,500 × g for 10 min, and the supernatant was treated with 150 μg of RNase per 10 ml at 37 °C for 30 min. Purification of the virus was performed on a CsCl gradient before dialysis against 140 mM NaCl, 25 mM Hepes, 5 mM MgCl_2_. The purified virus was stored at −80 °C^32,34^. IAV-WSN (H1N1) was grown in MDCK cells, and purified on a sucrose gradient^35,36^. The recombinant strain HSV-1-C12-CMV-GFP, referred to as HSV-1-GFP, was kindly provided by S. Efstathiou (University of Cambridge, Cambridge, UK)^37,38^. Virus was expanded on A549 cells (American Type Culture Collection) upon inoculation of supernatant from cells infected at 37 °C for 24 h. At 2 dpi, when strong CPE was visible, supernatant was collected, cleared from debris by centrifugation at 1,500 × g, 4 °C for 10 min, and centrifuged at 22,000 × g, 4 °C for 90 min to pellet virions. The pellet was agitated overnight in MNT buffer (30 mM MES, 100 mM NaCl, 20 mM Tris, pH 7.4) at 4 °C, resuspended and virus was purified by centrifugation through a nycodenz gradient of 25% and 30% w/v in MNT buffer at 20,000 rpm, 4 °C for 2 h. All purified viruses were stored at −80 °C. Virus titers were determined by TCID_50_ titration according to the Spearman-Kärber method.

### Prestwick chemical library, control compounds, and Z’-plates

The Prestwick Chemical Library (PCL) was obtained from Prestwick Chemical (Illkirch, France). Compounds for controls and validations were purchased from Abcam (Cambridge, UK), Cayman Chemical (Ann Arbor, USA), MedChemExpress (Monmouth Junction, USA), and Sigma-Aldrich (St. Louis, USA). Compounds were dissolved in DMSO at concentrations of 10, 50 or 100 mM. For each virus, a set of four microscopy grade 384-well plates (Becton Dickinson AG, Allschwil, Switzerland) containing the negative and positive controls (columns 1, 2 and 23, 24) as well as the PCL compounds (columns 3–22) were prepared at a final concentration of 1.25 μM using an Echo acoustic dispenser (Labcyte Inc., Indiana, USA) in four biological replicates. Positive controls were spotted at a concentration of 0.625 nM BafA1 for RV, 1.563 nM BafA1 for IAV, and 25 μM GCV for HSV, respectively. DMSO was spotted at equivalent volumes as negative control. This way, each screening plate contained 32 technical replicates of each positive and negative control, and 320 of PCL compounds in single technical replicates. To assess the assay quality, Z’-factors were determined for the optimized screening conditions for each virus. For this, 192 wells of each Z’-plate were spotted with the DMSO control and 192 wells with the positive control compounds. All plates were stored at −20 °C until use.

### High-throughput compound screening

Number of seeded cells, virus stock dilution, and incubation time were optimized prior to the execution of the compound screening. Determined parameters for the three viruses are summarized in Table 1. Cell seeding, virus infection, fixation, and staining were performed using a Matrix WellMate dispenser and the corresponding Matrix WellMate tubing cartridges (Thermo Fisher Scientific, Waltham, USA). Prior to use, plates were thawed for 30 min at room temperature (RT), briefly centrifuged, and 10 μl of PBS were added to each well to dissolve the compounds. Subsequently, cells were seeded in 60 μl culture medium, and incubated overnight at 37 °C. On the next day, virus was added in a volume of 10 μl culture medium per well. Plates were incubated for 24–48 h, before they were fixed with 26.6 μl 16% paraformaldehyde (PFA) and 4 μg/ml Hoechst 33342 (Sigma-Aldrich, St. Louis, USA) in PBS for 1 h at RT, followed by three washings with PBS, and final addition of 0.02% NaN_3_ in PBS for storage at 4 °C. For IAV, cells were incubated with 50 μl of NH_4_Cl for 10 min at RT, followed by treatment with 0.25% Triton-X-100 in PBS for 5 min. After three washes with PBS, cells were blocked with 1% BSA (Sigma-Aldrich, St. Louis, USA) for 30 min at RT. Staining with primary mouse monoclonal anti-IAV NP antibody HB-65 was carried out at a dilution of 1:15 in PBS supplemented with 1% BSA in a volume of 30 μl per well for 1 h at 4 °C. Cells were washed three times with PBS and incubated with secondary antibody anti-mouse Alexa-Fluor 488 (Thermo Fisher Scientific, Waltham, USA) in a 1:1000 dilution in PBS supplemented with 1% BSA and 4 μg/ml Hoechst 33342 in a volume of 30 μl for 1 h at RT. After three washes with PBS, 0.02% NaN_3_ in PBS was added to the cells for storage at 4 °C. For all steps, standard bore tubing cartridges were used, and tubings were rinsed with 125 ml autoclaved ddH_2_O and PBS before and after use, and autoclaved before next use.

### Imaging

Images of individual wells were acquired with the DAPI channel (nuclei stained with Hoechst 33342) and the FITC channel (viral GFP or NP). The plates for RV and IAV were imaged at UZH and EPFL, while plates for HSV were imaged at UZH only. At UZH, we used an IXM-C automated inverted epifluorescence microscope (Molecular Devices, San Jose, USA) at 4x magnification^39^. At EPFL, plates were imaged using an IN CELL 2200 automated high-throughput fluorescence microscope (GE Healthcare, Chicago, USA)^5^.

### Image analysis and data processing

Five main parameters were scored using the software Plaque2.0^26^ to quantify the infection phenotype: number of nuclei, number of infected nuclei, infection index (ratio between total number of nuclei and number of infected nuclei), total virus intensity, and number of plaques (infection foci). Each of the institutions optimized Plaque2.0 parameters according to the acquired data, and all read-outs can be found in the Plaque2.0 output files ImageData.csv and ObjectData.csv. The obtained results were then processed in R version 4.0.2^40^ at UZH, and in KNIME version 3.5.3^41^ together with the EPFL-BSF in-house LIMS at EPFL. For each of the four biological replicates of the 1,280 PCL compounds as well as for the 32 technical replicates on 16 biological replicates of the positive and negative controls, the mean scores for the five main read-outs were calculated. Each compound score was then normalized by the mean score of the DMSO negative control for each plate individually. All compounds within a threshold of μ_-_ (mean of the negative control) ± 2σ (SD of the negative control) for number of nuclei were filtered for toxicity, and beyond a threshold of μ_+_ ± 1.5–3σ for the infection scores (number of infected nuclei, infection index, number of plaques or integrated GFP intensity) for efficacy.

### Z’-factor calculation

According to Eq. 1, Z’-factors^42^ were determined using R version 4.0.2:

1 $${Z}^{{\prime} }{\rm{=\; 1}}-\frac{\left({{\rm{3\sigma }}}_{+}+{{\rm{3\sigma }}}_{-}\right)}{\left|{\mu }_{+}-{\mu }_{-}\right|}$$

where σ_+_ is the SD of the positive control, σ_-_ is the SD of the negative control, μ_+_ the mean of the positive control and μ_-_ the mean of the negative control.

### Z-score calculation

Z-scores were computed in Python version 3.7.1^43^ according to Eq. 2:

2 $${\rm{Z}}=\frac{{\rm{X}}-{\rm{\mu }}}{{\rm{\sigma }}}$$

where X is the plaque number or infection index of the compound, and µ and σ are the mean and SD of the negative control of the plate where the compound was spotted.

### PrestoBlue toxicity assay

While toxicity of the PCL compounds on infected cells was assessed with the screening read-out ‘total number of nuclei’, toxicity of the compounds on uninfected A549 and HOG cells was determined using the PrestoBlue Cell Viability reagent (Thermo Fisher Scientific, Waltham, USA). PrestoBlue^44,45^ indicates the viability of cells via conversion of non-fluorescent resazurin to a strongly fluorescent resorufin during cellular respiration. Specifically, cells were treated with the compounds in duplicates for 24 h (A549) or 48 h (HOG) at the same concentrations and cell densities as used in the screening. Then, PrestoBlue was added to each well to a final concentration of 10%, and the cells were incubated at 37 °C (A549) or 33.5 °C (HOG) for 1 h. A multi-well plate-reader (Tecan Infinite F500, Tecan, Männedorf, Switzerland) was used to measure fluorescence intensity at an excitation of 560/10 nm and emission at 590/10 nm. As positive control for cytotoxicity, Doxorubicin hydrochloride (Prestwick Chemical, Illkirch, France) at a final concentration of 10 μM was used, with corresponding volume of DMSO as negative control. Data processing and statistical validation were performed using the EPFL-BSF in-house Laboratory Information Management System (LIMS). Raw values were normalized to the positive (=1) and negative (=0) control, before averaging. Compounds were classified as toxic if the normalized value for both replicates was higher than the average + 3σ (standard deviation, SD) of the DMSO negative control for the same plate.

### Phospolipidosis assay

The phospolipidosis assay was performed as previously described^31^. Briefly, 15,000 A549 ATCC cells were seeded in each well of a 96-well imaging plate and grown overnight in DMEM supplemented with 10% FCS and 1x NEAA. On the next day, medium was replaced with DMEM supplemented with 10% FCS, 1x NEAA, 7.5 µM NBD-PE (Thermo Fisher Scientific, Waltham, USA), cells treated with compounds at 37 °C for 24 h, stained with a solution of DMEM, 100 mM sodium pyruvate, 200 mM L-Glutamine, 10% FCS, 1x NEAA, 10 µg/mL Hoechst 33342, and 2 µM Ethidium homodimer-2 (EthD-2, Thermo Fisher Scientific, Waltham, USA) at 37 °C for 30 min. Microscopy images were acquired at ImageXpress Micro confocal (Molecular Devices). For image analyses, nuclear area was expanded to a diameter of 50 µm and the integrated NBD-PE fluorescence measured in the EthD-2-negative, intact cells. Drug-induced phospholipidosis (DIPL) scores were normalized to the mean integrated intensity of the positive control Amiodarone used at 10 µM dosage.

## Data Records

### Data structure and repositories

Data was deposited to the Image Data Resource (IDR) (https://idr.openmicroscopy.org) under accession numbers idr0128 (IAV)^27^, idr0129 (RV)^28^ and idr0130 (HSV)^29^. The data available for download at the IDR consists of information acquired during screen optimization, screening procedure, and analysis. Fig. 2a,c depict the data structure for download. Raw images, analysis files, parameters used for Plaque2.0, and the code for hit filtering in R can be downloaded from the IDR, following the instructions from idr.openmicroscopy.org/about/download. The data can also be viewed on the IDR web client, structured as outlined in Fig. 2b,d, at idr.openmicroscopy.org/webclient. A list of annotated PCL compounds, and raw and scored screening data are available on figshare^46^.

### Data sets and file types

As outlined in Fig. 2a,c, we provide three data sets for each virus for download:

- *1-prePlates* contains layouts (.csv), images (.tif), Plaque2.0 image analysis parameters (.mat) and results (.csv) for the assay stability test plates performed at UZH prior to Z’-factor plates (*preZ*) and the screen (*preScreen*).
- *2-ZPlates* contains layouts (.csv), images (.tif), Plaque2.0 image analysis parameters (.mat) and results (.csv) for the two Z’-factor plates *a* and *b* as imaged and analyzed at UZH (*Data_UZH*) and EPFL (*Data_EPFL*). For HSV, data was acquired at UZH only.
- *3-Screen* contains layouts (.csv), images (.tif), Plaque2.0 image analysis parameters (.mat) and results (.csv) for the 16 screening plates (four biological replicas *1*–4, each consisting of a set of four subset plates *A - D*) as imaged and analyzed at UZH (*Data_UZH*) and EPFL (*Data_EPFL*). For HSV, data was acquired at UZH only. Moreover, *Analysis* contains the Plaque2.0 batch processing and hit filtering pipeline (*batchAnalysis.R*) used by UZH. *Analysis* also contains the PrestoBlue raw results (.csv) for toxicity in absence of infection.

Fig. 2b,d depict the structure of the data for viewing in the IDR web client:

- *idr0128-study.txt*, *idr0129-study.txt,* and *idr0130-study.txt* summarize the overall study and the screens that were performed.
- *screenA* contains the assay stability test plates performed at UZH prior to Z’-factor plates (*preZ*) and the screen (*preScreen*). *idr0128-screenA-library.txt, idr0129-screenA-library.txt,* and *idr0130-screenA-library.txt *provide thorough information on the tested compounds including PubChem identifiers and their plate layout. *idr0128-screenA-processed.txt, idr0129-screenA-processed.txt,* and *idr0130-screenA-processed.txt* present the results of the Plaque2.0-based image analysis. *idr0128-screenA-mean.txt, idr0129-screenA-mean.txt,* and *idr0130-screenA-mean.txt* summarize the infection scores per pre plate.
- s*creenB* contains the assay quality test plates (Z’-factor plates *a* and *b*) performed at UZH. *idr0128-screenB-library.txt, idr0129-screenB-library.txt,* and *idr0130-screenB-library.txt* provide thorough information on the tested compounds including PubChem identifiers and their plate layout. *idr0128-screenB-processed.txt, idr0129-screenB-processed.txt,* and *idr0130-screenB-processed.txt *present the results of the Plaque2.0-based image analysis. *idr0128-screenB-mean.txt, idr0129-screenB-mean.txt,* and *idr0130-screenB-mean.txt *summarize the infection scores per Z’-factor plate.
- *screenC* contains the assay quality test plates (Z’-factor plates *a* and *b*) performed at EPFL. *idr0128-screenC-library.txt* provides thorough information on the tested compounds including PubChem identifiers and their plate layout. *idr0128-screenC-processed.txt* presents the results of the Plaque2.0-based image analysis. *idr0128-screenC-mean.txt* summarizes the infection scores per Z’-factor plate. This data was only acquired for IAV.
- *screenD* (*idr0129-screenC* for RV and *idr0130-screenC* for HSV) contains the PCL screening plates (in replicates *1* to *4*, consisting of subset plates *A* to *D* for RV and IAV; and replicates 1 to 3, consisting of subset plate *A* to *C* for HSV, respectively) performed at UZH. *idr0128-screenD-library.txt* provides thorough information on the tested compounds including PubChem identifiers and their plate layout. *idr0128-screenD-processed.txt* presents the results of the Plaque2.0-based image analysis. *idr0128-screenD-filtered.txt* summarizes the infection scores per compound and indicates if it was identified as hit.
- *screenE* (*idr0129-screenD* for RV) contains the PCL screening plates (in replicates 1 to 4, consisting of subsets *A* to *D*) performed at EPFL. *idr0128-screenE-library.txt* provides thorough information on the tested compounds including PubChem identifiers and their plate layout. *idr0128-screenE-processed.txt* presents the results of the Plaque2.0-based image analysis. *idr0128-screenE-filtered.txt* summarizes the infection scores per compound and indicates if it was identified as hit. This data was not acquired for HSV.

## Technical Validation

### Assay stability

High assay stability and reproducibility were ensured by optimization of the screening procedure. The optimization of the screening pipeline included liquid handling, cell seeding, virus inoculum, infection time, controls, exposure times and other parameters during imaging, and parameters for image analysis. All compounds and reagents were prepared as large batches from a single lot and split to single-use aliquots. Virus stock dilutions were tested regularly for efficacy, and adjusted if necessary. Pre-plates were assessed prior to every experiment to control for the functionality of the pipeline.

### Assay quality

To assess the accuracy of the screening pipeline, we determined Z’-factors for the five parameters that we used for scoring the infection phenotype. Two Z’-factor plates were imaged and analyzed independently at UZH and EPFL for RV and IAV, and only at UZH for HSV (Table 2). For RV, mean 3σ Z’-factors ranged between 0.49–0.53 for the read-outs numberOfInfectedNuclei, infectionIndex, numberOfPlaques, and totalVirusIntensity, scoring excellent. For IAV, mean 3σ Z’-factors ranged between 0.13–0.37 for the same readouts, scoring good. HSV read-outs ranged between 0.15–0.37 for numberOfInfectedNuclei, infectionIndex, numberOfPlaques, scoring good. The mean 3σ Z’-factor for totalVirusIntensity was 0.1, rendering the parameter unsuitable for read-out. For all three viruses, mean 3σ Z’-factors of the Z’-factor plates as well as for the screening plates for the parameter numberOfNuclei were negative, confirming that there was no significant cell death caused by the viruses or control compounds. The 3σ Z’-factors were determined for each of the 16 screening plates per virus, and averaged. The mean 3σ Z’-factors of the screening plates for RV and HSV were in the range of 0.36–0.66, scoring good to excellent, and the Z’-factors of the screening plates for IAV ranged from 0.1–0.37, scoring good.

### Independent analysis and filtering

Two independent research groups, at UZH and EPFL, performed acquisition of plate images, image analysis, and data processing (Fig. 1) for the RV and IAV screens. We compared the variance between the measurements of the two institutions by correlating the compound ranks based on the Z-scores of each compound. For the computed parameters number of plaques and infection index the Pearson correlation coefficients were the following: IAV (infection index) = 0.922, IAV (number of plaques) = 0.818, RV (infection index) = 0.852, RV (number of plaques) = 0.407, indicating good to excellent correlation (Supplementary Fig. 3). Since we achieved high reproducibility and correlation of results from the two institutions in the screenings of these viruses, we decided to further perform the imaging and analysis of HSV plates only at UZH. Both analysis pipelines confirmed high assay quality (Tables 2 and 3). Compounds that were identified as toxic in uninfected cells (PrestoBlue assay) were excluded during hit filtering. This resulted in the following top virus inhibitory hits: 5-Azacytidine (Prestw-338) for RV, Clotrimazole (Prestw-267) for IAV, and Raltitrexed (Prestw-1793) for HSV-1 (Table 1).

## Usage Notes

Five parameters were used to score the infection phenotype of each well: the number of nuclei (numberOfNuclei), number of infected nuclei (numberOfInfectedNuclei), the ratio between number of infected and total nuclei (infectionIndex), the number of multi-round infection foci termed plaques (numberOfPlaques), and the extend of viral GFP reporter expression as integrated GFP intensity (totalVirusIntensity).

### Infection scoring using the Plaque2.0 GUI

A detailed manual for Plaque2.0-based infection phenotype scoring is available at http://plaque2.github.io/. No MATLAB license is necessary.

The following settings should be used:

Input/Output: *Processing Folder*: Path to folder containing the images (e.g. *idr0128/3-Screen/Data_EPFL/Screen/ BSF019243_1A*).

*filename pattern* Data_UZH:.* (?<wellName>[A-Z][0-9]*)_(?<channelName>w[0–9]*).TIF

*filename pattern* Data_EPFL:.* (?<wellName>[A-Z] - [0–9]+)[(]fld 1 wv (?<channel>[A-Z]{4}).*.tif

*Plate name*: Name of the plate to be analyzed (e.g. *BSF019243_1A*)

*Result Output Folder*: Path to the results folder in the respective Data folder (e.g. *idr0128/3-Screen/Data_EPFL/Results*).

Stitch: Stitching of the images is not necessary, since every 384-well is imaged in a single site. Do not activate the tab.

Mask: *Custom Mask File*: Path to the manually defined mask file (e.g. *idr0128/3-Screen/Data_UZH/Parameters*). Well masking is optional and was not performed by EPFL.

Monolayer: *Channel*: Nuclei were imaged in channel 1.

Plaque: *Channel*: Viral GFP reporter signal or NP antibody signal, respectively, was imaged in channel 2.

Infection scoring using the Plaque2.0 GUI

Please refer to the comments of the code AntiVir_batchprocessing.m for usage instructions.

## Supplementary information

Supplementary Information

## Data Availability

Plaque2.0 batch image analysis for infection scoring was performed by MATLAB (version R2016b, The MathWorks, Natick, USA) script *AntiVir_batchprocessing.m* used by UZH for image analysis is provided at IDR under *idr0130/3-Screen/Analysis*. It is based on the Plaque2.0 software available on GitHub: https://github.com/plaque2/matlab under GPLv3 open source license. To batch analyze the virus screening data by Plaque2.0, fork or download the Plaque2.0 AntiVir code from GitHub: https://github.com/plaque2/matlab/tree/antivir. Place the *AntiVir_batchprocessing.m* file from *idr130/3-Screen/Analysis* into the *Plaque2/matlab* folder and follow the instructions in *AntiVir_batchprocessing.m*. A MATLAB license is required. Hit filtering using R was performed in R (version 4.0.2.) script *AntiVir_hitfiltering* used by UZH for data processing and hit filtering is provided at IDR under *idr130/3-Screen/Analysis*.
