# Supplementary material for: High-content, arrayed compound screens with rhinovirus, influenza A virus and herpes simplex virus infections
Source: Sci Data. 2022 Oct 8;9:610. doi: 10.1038/s41597-022-01733-4 (PMC9547564; doi:10.1038/s41597-022-01733-4)
Supplement: Supplementary file 1 — Supplementary Information [file 41597_2022_1733_MOESM1_ESM.pdf]

Supplementary Information

High-content, arrayed compound screens with rhinovirus, influenza A virus and herpes simplex virus infections

Dominik Olszewski[1,*], Fanny Georgi[1,*], Luca Murer[1], Vardan Andriasyan[1], Fabien Kuttler[2], Anthony Petkidis[1], Robert Witte[1], Artur Yakimovich[3,6], Lucy Fischer[1], Alina Rozanova[1,4], Yohei Yamauchi[4, 5], Gerardo Turcatti[2], Urs F. Greber[1]

Affiliations

1 Department of Molecular Life Sciences, University of Zurich (UZH), Winterthurerstrasse 190, 8057 Zurich, Switzerland

2 Biomolecular Screening Facility, School of Life Sciences, Ecole Polytechnique Fédérale (EPFL) de Lausanne, Station 15, Lausanne 1015, Switzerland

3 Center for Advanced Systems Understanding (CASUS), Helmholtz Center Dresden-Rossendorf, Untermarkt 20, 82026 Görlitz, Germany

4 University of Bristol, Bristol BS8 1TH, United Kingdom,

5 present address: Institute of Pharmaceutical Sciences, ETH Zurich, Vladimir-Prelog-Weg 1-5/10, 8093 Zurich, Switzerland

6 Bladder Infection and Immunity Group (BIIG), Department of Renal Medicine, Division of Medicine, University College London, Royal Free Hospital Campus, London NW3 2PF, United Kingdom

***these authors contributed equally**

corresponding author(s): Urs F. Greber (urs.greber@mls.uzh.ch)

Table of Contents

Supplementary Figure 1: Validation of best hit compounds. III

Supplementary Figure 2: Drug-induced phospholipidosis (DIPL) of selected candidate drugs. V

Supplementary Figure 3: Z-score correlation of compound ranks for the IAV and RV screens based on UZH and EPFL analyses. VI

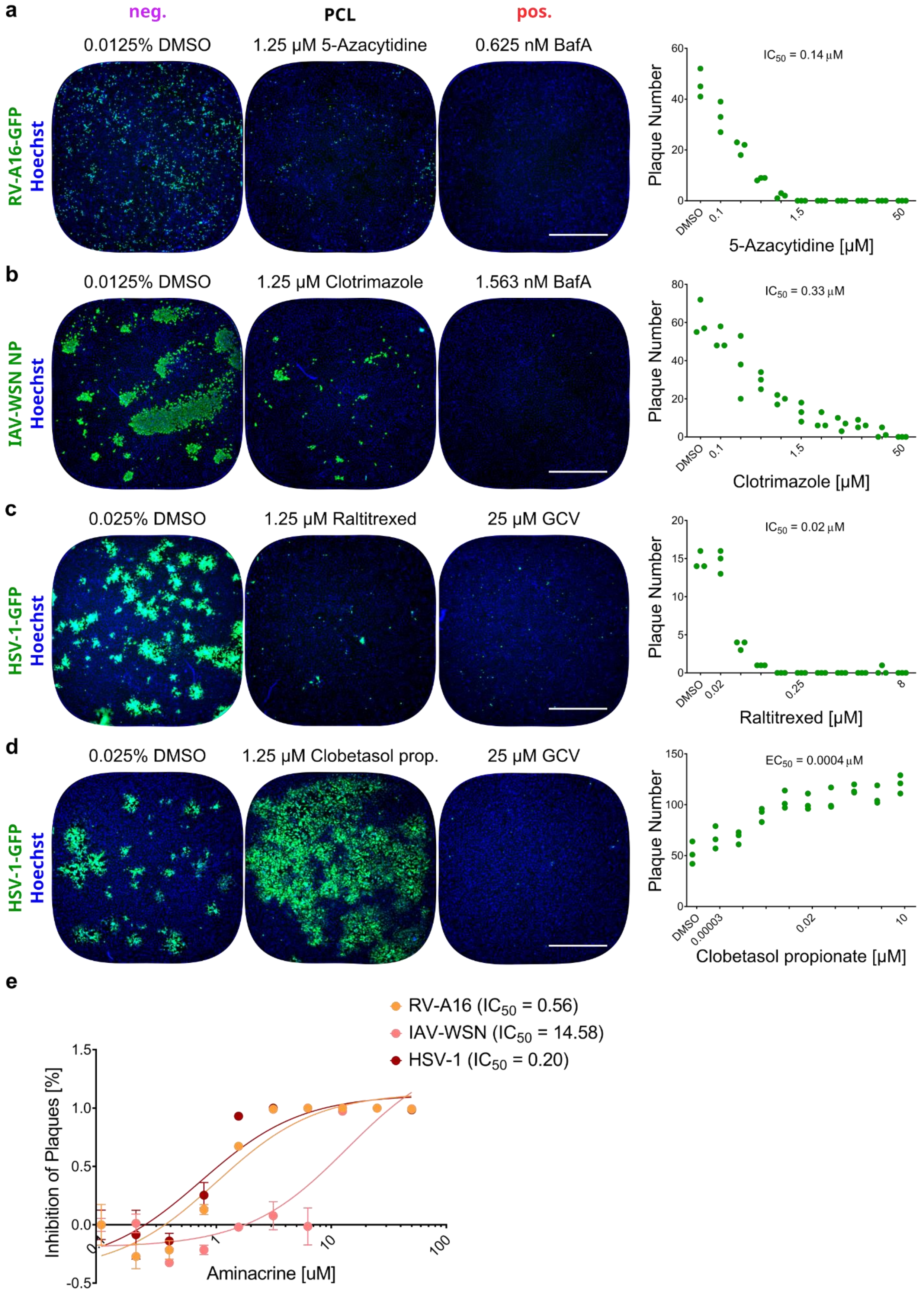

Supplementary Figure 1: Validation of best hit compounds.

(a-d) Exemplary images from the screen along with the respective control compounds are shown on the left side. Top compounds were tested in follow-up experiments for effects on viral infections. All compounds were titrated in triplicates on a cell monolayer in a 96-well plate format prior to cell seeding and infection. Cells were infected with RV for 48 h, IAV for 24 h, and HSV-1 for 26 h, PFA fixed, stained and imaged. Images were analyzed for number of plaques, and effective concentrations of the compounds were determined. All top hits had effective concentrations in the sub- to high nanomolar range. Scale bar represents 2 mm. (e) Inhibition of viral plaque formation by Aminacrine. Aminacrine was added to cells at one day post seeding together with virus. Inocula amounted to 30 FFU of RV, 60 FFU of IAV, and 100 FFU of HSV-1, and were left on cells for 24 h (IAV, HSV) or 48 h (RV). Inhibition of infection could also be shown for IAV, even though Aminacrine did not score as a top hit in the IAV screen. PCL = Prestwick Chemical Library.

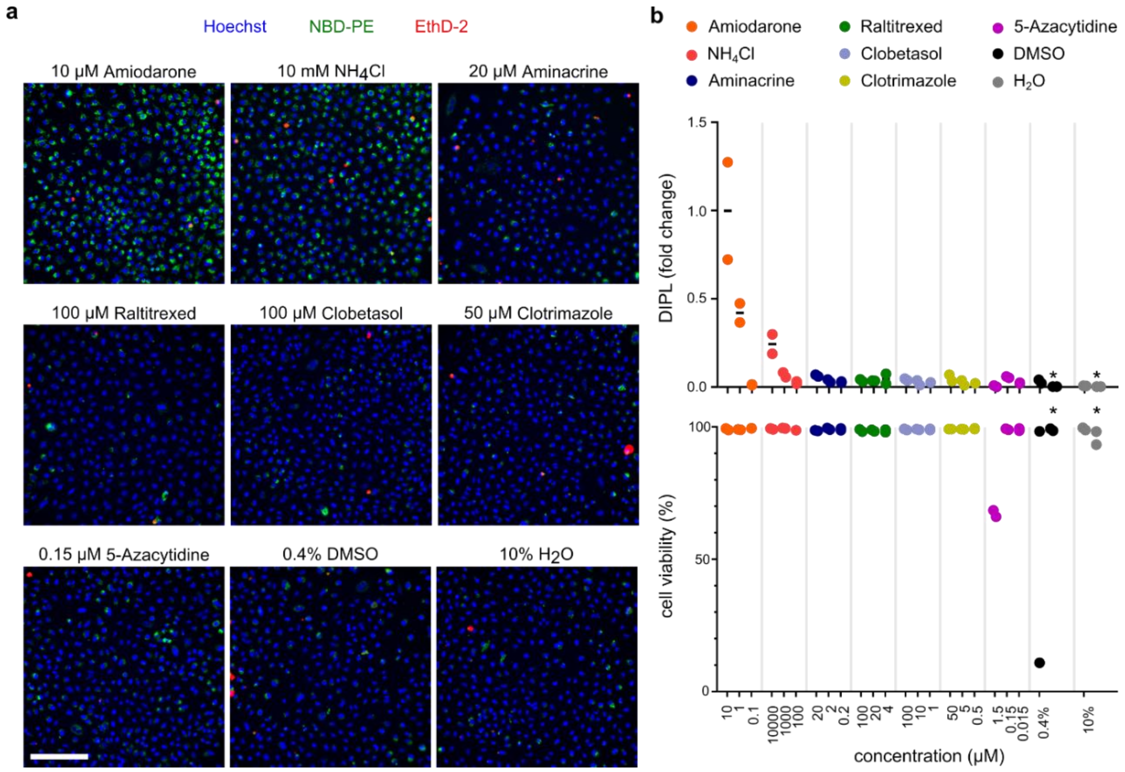

Supplementary Figure 2: Drug-induced phospholipidosis (DIPL) of selected candidate drugs.

a) Example images of A549 ATCC cells treated with the indicated drugs. Scale bar = 200 µm. (b) Quantification of DIPL and cell viability. DIPL was assessed by the integrated cellular fluorescence of NBD-PE and normalized to the mean of the amiodarone treatment. Cell viability was assessed by EthD-2 exclusion. Asterisk indicates no addition of NBD-PE.

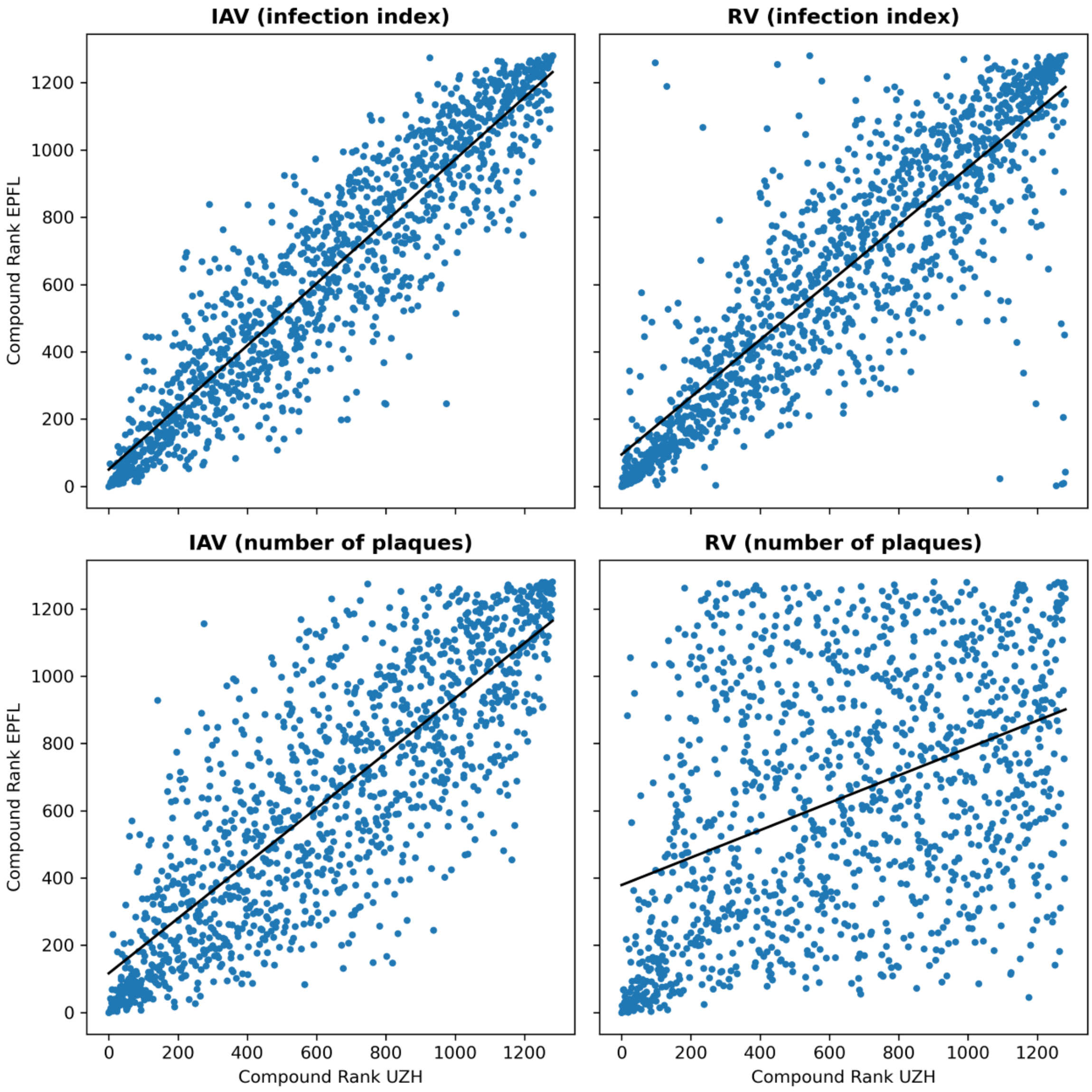

Supplementary Figure 3: Z-score correlation of compound ranks for the IAV and RV screens based on UZH and EPFL analyses.

Mean Z-scores of all compounds were calculated, and compounds were ranked according to their inhibitory effect (lowest number equals rank 1). For data analyzed by UZH and EPFL, compound ranks (blue dots) and linear regression (black line) were plotted.
